# 2‐[ ^18^F] FDG PET/CT in Rapid Late‐Onset Multiple Acyl‐CoA Dehydrogenase Deficiency: A Case Report

**DOI:** 10.1002/jmd2.12469

**Published:** 2025-02-12

**Authors:** Astrid Høj, Sonja Holm‐Yildiz, Thomas Krag, Danijela Dejanovic, Thomas van Overeem Hansen, Morten Dunø, Mette Cathrine Ørngreen, John Vissing, Nicoline Løkken

**Affiliations:** ^1^ Department of Neurology, Copenhagen Neuromuscular Centre Copenhagen University Hospital, Rigshospitalet Copenhagen Denmark; ^2^ Department of Clinical Physiology, Nuclear Medicine, and PET, Rigshospitalet University of Copenhagen Copenhagen Denmark; ^3^ Department of Clinical Genetics, Molecular Genetic Laboratory, Rigshospitalet University of Copenhagen Copenhagen Denmark; ^4^ Department of Clinical Medicine University of Copenhagen Copenhagen Denmark; ^5^ Department of Paediatrics and Adolescent Medicine Copenhagen University Hospital, Rigshospitalet Copenhagen Denmark

**Keywords:** FDG PET/CT, lipid myopathy, MADD, metabolic myopathy, multiple acyl coenzyme‐A dehydrogenase deficiency, muscle biopsy

## Abstract

Multiple acyl‐CoA dehydrogenase deficiency (MADD) is a rare inborn metabolic myopathy affecting fat and protein metabolism. Patients with late‐onset MADD typically present with exercise intolerance and muscle weakness. We present a patient with an acute, very late‐onset symptom debut at 52 years of age. Over 5 months, the patient deteriorated from asymptomatic to almost complete loss of ambulation. He had a substantial weight loss, head‐drop, progressive proximal limb and chewing weakness. Due to the rapid progression, amyotrophic lateral sclerosis, myositis, myasthenia gravis and a paraneoplastic syndrome in relation to underlying malignancy were considered first. A 2‐[^18^F] FDG PET/CT scan was performed to exclude a paraneoplastic syndrome. The scan revealed diffuse and symmetric, pathologically high 2‐[^18^F] FDG‐uptake in the patient's neck, shoulder, and paravertebral muscles, which was later suggested as a sign of a metabolic myopathy. Muscle biopsy (Oil Red O staining) and acylcarnitine profile (elevated C5‐C18 acylcarnitines) findings suggested MADD, which was confirmed by genetic analysis showing biallelic variants in the *ETFDH* gene (c.1763A>G, p.(His588Arg); c.897G>A, p.(Leu299=)). After 1 month of dietary intervention and daily diet supplements (riboflavin 400 mg TID, levocarnitine 1 g TID, Q10 150 mg qD in two doses), the patient had almost recovered to his habitual level. A posttreatment muscle biopsy showed less disrupted ultrastructure of the myofibers. We learned from this case of rapid and late‐onset MADD that 2‐[^18^F] FDG PET/CT, with diffuse and symmetric 2‐[^18^F] FDG‐uptake in skeletal muscle, can be valuable in clarifying this rare diagnosis.

**Summary**

- A case of very late‐onset Multiple Acyl‐CoA Dehydrogenase Deficiency (MADD) with debut at 52 years showed rapid progression to severe muscle weakness.
- 2‐[^18^F] FDG PET/CT scan revealed diffuse and symmetric 2‐[^18^F] FDG uptake in skeletal muscles suggestive of a metabolic myopathy.
- Muscle biopsy, acylcarnitine profiling and genetic testing identifying pathogenic ETFDH variants confirmed the MADD diagnosis.
- Treatment with riboflavin, levocarnitine, and coenzyme Q10 led to near‐complete recovery within one month.
- This case highlights the potential diagnostic utility of 2‐[^18^F] FDG PET/CT in rapidly progressing metabolic myopathies.

## 1 Introduction

Multiple acyl‐CoA dehydrogenase deficiency (MADD, OMIM# 231680) is a rare, autosomal recessive metabolic myopathy affecting fat and protein metabolism. MADD is caused by pathogenic variants in the genes *ETFA*, *ETFB*, or *ETFDH*—most commonly in the *ETFDH* gene [1]. These lead to defects in the electron transfer flavoprotein, resulting in dysfunctional mitochondria and lipid storage myopathy [2]. MADD is a very rare disease with an incidence of 1:250.000 [1]. Clinically, MADD can be divided into three types according to the age of onset and the presence or absence of congenital anomalies. Type 1 is neonatal onset with congenital anomalies, type 2 is neonatal onset without congenital anomalies, and type 3 is late‐onset [1]. Late‐onset MADD is the most common type, typically diagnosed at late teens to early twenties [1, 3]. The clinical presentation is very heterogeneous, especially in the late‐onset type. The symptoms are mainly comprised of fluctuating or progressive muscle weakness, usually in the proximal limbs, acute metabolic decompensations, or both muscular and metabolic symptoms [1, 3]. Typically, there is a delay between the onset of symptoms and diagnosis, with a mean of 3.9 years [4]. Here, we report a male who did not experience symptoms until 52 years of age, where over a short time span, he deteriorated from an active lifestyle to only being able to walk a few meters with assistance. The purpose of this case is to report the diagnostic work‐up of this very late‐onset MADD case, and to highlight and discuss the potential use of 2‐deoxy‐2‐[^18^F] fluoro‐D‐glucose positron emission tomography/computed tomography (2‐[^18^F] FDG PET/CT) imaging in this process.

## 2 Clinical Findings, Diagnosis, Treatment, and Outcome

The patient was on disability pension due to a back injury but was otherwise living an active life until debut of symptoms. The patient deteriorated over 5 months from an active lifestyle with workouts three to four times a week to almost complete loss of ambulation. He was bed‐bound for most of the day and could only walk few meters with assistance. Initially, the patient had a workup for gastrointestinal cancer due to signs of malignancy (weight loss and fatigue) in combination with diarrhea, abdominal pain, and increased alanine transaminase (ALT). After a negative colonoscopy and thoracic and abdominal CT scan, the patient was referred to our neuromuscular clinic. He presented at our clinic with an unintended 15 kg weight loss (17% of his body weight), head‐drop, progressive proximal limb weakness, chewing weakness, difficulty swallowing, and diarrhea (6–15 times daily). His gait was broad‐based and with a hyper‐lordotic posture. Muscle strength evaluation using the Medical Research Council (MRC) scale [5] revealed a symmetric muscle weakness. The patient displayed muscle weakness (4+ strength) on neck extension, shoulder abduction, elbow extension, finger extension, finger abduction, hip extension, hip adduction, hip abduction, and hip extension. He had normal (5) strength in the remaining muscle groups.

**FIGURE 1:**
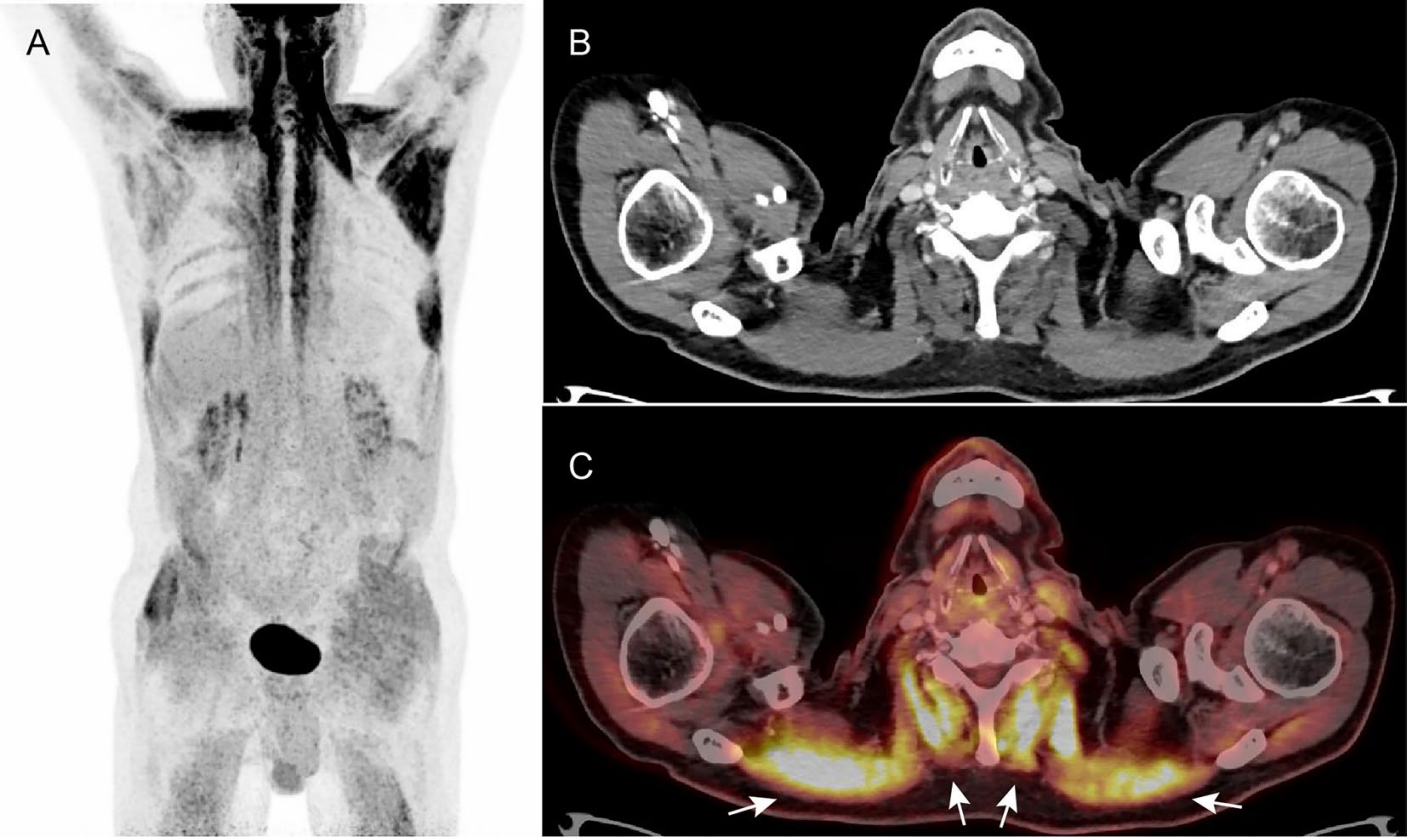
2‐[^18^F] FDG PET/CT scan of the patient before treatment. (A) Maximum intensity projection shows markedly high and symmetrical 2‐[^18^F] FDG uptake in the neck and shoulder muscles as well as high uptake in muscles of the gluteal region and both arms and legs. (B, C) CT and the fused PET/CT image demonstrates high 2‐[^18^F] FDG uptake in the neck muscles (arrows).

Several diagnoses were considered including amyotrophic lateral sclerosis (ALS), myositis, myasthenia gravis, underlying malignancy and/or a paraneoplastic syndrome, which led to a broad diagnostic workup. This included blood samples (including creatine kinase (CK), myoglobin, angiotensin converting enzyme, erythrocyte sedimentation reaction, polymyositis antibodies, and paraneoplastic antibodies), lumbar puncture, electromyography (EMG), and a muscle biopsy. Although not the most likely diagnosis, but in order to rule out malignancy/paraneoplastic syndrome, a 2‐[^18^F] FDG PET/CT scan was performed. The scan revealed diffuse and symmetric, pathologically high 2‐[^18^F] FDG‐uptake in the neck, shoulder, and paravertebral muscles (Figure 1). The increased uptake was found in the patient's weak muscles. Findings were retrospectively considered consistent with a metabolic myopathy.

**FIGURE 2:**
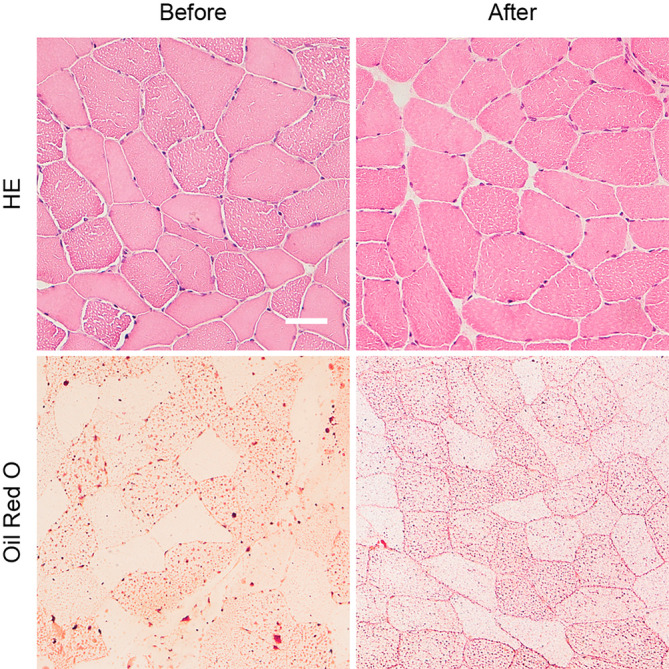
Histology of vastus lateralis muscle before and after treatment. Hematoxylin and Eosin (HE) stained muscle section clearly demonstrate the lipid filled myofibers with a sieve‐like pattern before treatment. An HE stained section from a biopsy taken after treatment has commenced, demonstrates a less clear somewhat finer pattern of lipid accumulation. This difference is also seen in the Oil Red O stain for lipid, where lipid accumulates in larger vesicles before treatment compared with the much finer even spread of lipid in muscle after treatment. Bar is 50 μm.

The muscle biopsy was obtained from the vastus lateralis muscle using the Bergström's method [6]. Hematoxylin and Eosin (HE) staining for general histopathology and Oil Red O stain for lipid demonstrated a coarser accumulation of lipid in muscle fibers with larger accumulations, suggesting a lipid storage myopathy, such as MADD (Figure 2). In MADD, acylcarnitine (AC) profile typically shows elevation of several AC chains [4]. The presented case had considerably elevated ACs with elevation of C5‐C18 increasing the likelihood of an MADD diagnosis (Table 2). Despite the severe loss of muscle mass, the patient surprisingly had a normal CK level ranging from 162 to 282 U/L (reference range 40–280 U/L). CK‐levels can be normal in asymptomatic patients. However, in symptomatic patients, it can be markedly elevated (20 000 U/L < in one patient [2]), and thus the normal CK level found in this case was unusual. Lastly, genetic analysis identified biallelic missense and synonymous variants in the *ETFDH* gene (NM_004453.4). In silico protein analysis of the missense variant (c.1763A>G, p.(His588Arg)) indicated a deleterious effect (CADD score 25.6, REVEL score 0.906). The variant had previously been observed in an individual with MADD (PMID 24357026) and is reported once in ClinVar as pathogenic. The synonymous variant (c.897G>A, p.(Leu299=)), known in gnomAD and reported once in ClinVar as likely benign. In silico analyses did however indicate possible introduction of a cryptic acceptor splice site in exon 8. Therefore, RNA analysis was performed using RNA from muscle. Reverse‐transcriptase polymerase chain reaction (RT‐PCR), semi‐quantitative capillary electrophoresis and nanopore sequencing, revealed a major transcript (40%–50% of the total number of transcripts) that leads to skipping of exon 8, resulting in an in‐frame deletion of 47 amino acids (p.(Leu278_Val324del)). The abovementioned tests altogether confirmed the MADD diagnosis. The remaining diagnostic assessments either confirmed the diagnosis or excluded the differential diagnosis, with the EMG exam showing signs of myopathy and not ALS and repetitive stimulation showed no signs of myasthenia gravis. Furthermore, the lumbar puncture and remaining laboratory studies were normal.

The patient received standard treatment for MADD [7] (oral treatment of riboflavin 400 mg TID, levocarnitine 1 g TID, Q10 150 mg qD in two doses), dietary changes to a high‐carbohydrate, low‐fat, low‐protein diet along with physiotherapy. The patient reported a significant effect quickly after initiation of treatment. Before and after treatment, we evaluated muscle strength with isokinetic dynamometry (Biodex, Shirley, NY) and physical function with 10‐m walking test and timed up‐and‐go (Table 1). We found great improvement in all tests after only 1 month of treatment (Table 1). Notably, the patient could walk without crutches and was even able to do light running. The muscle strength improved 1.4‐fold in the shoulder abduction and adduction, and in hip flexion and extension by 1.2‐fold and 3.2‐fold, respectively.

**TABLE 1:** Isokinetic muscle strength measured with stationary dynamometry.

Muscle strength	Pretreatment	CV%	1‐month posttreatment	CV%	2‐months posttreatment	CV%
Shoulder abduction	33.3 Nm	2.5%	47.4 Nm	0.6%	56.7 Nm	1.1%
Shoulder adduction	45.2 Nm	5.9%	62.0 Nm	1.0%	63.0 Nm	2.0%
Hip flexion	62.5 Nm	5.7%	76.2 Nm	2.7%	115.4 Nm	2.6%
Hip extension	26.5 Nm	11.8%	84.6 Nm	9.2%	84.2 Nm	0.5%

Walking test	Pretreatment	1‐month posttreatment	2‐months posttreatment
10‐m walking test	7.4 s	4.0 s	2.8 s
Timed up‐and‐go	9.5 s	7.0 s	6.0 s

Abbreviations: CV: coefficient of variation; Nm: Newton‐meter; s: seconds.

The patient's acylcarnitine profile improved with the ratio between free and total acylcarnitines increasing from 41% to 64% (reference range 65%–87%) (Table 2). Seventeen months post treatment initiation, there are still elevations in acylcarnitines C5‐C18, though some have moved closer to reference values.

**TABLE 2:** Acylcarnitine profile.

Plasma values (μmol/L)	Pre‐treat.	17 mo. post‐treat.	Ref. range
Carnitine (C0)	10	31	20–49
Acetylcarnitine (C2)	**2.0**	5.9	2.8–14
Propionylcarnitine (C3)	**0.09**	0.26	0.15–0.91
Isobutyrylcarnitine (C4)	0.18	0.07	0.02–0.49
Butyrylcarnitine (C4)	0.24	0.18	0.03–0.39
3‐Hydroxybutyrylcarnitine (C4OH)	0.01	0.00	0–0.05
Malonylcarnitine (C3DC)	0.00	**0.01**	0–0
Succinylcarnitine (C4DC)	0.00	0.01	0–0.03
Methylmalonylcarnitine (C4DC)	0.00	0.01	0–0.01
Pivaloylcarnitine (C5)	0.00	0.00	0–0
2‐Methylbutyrylcarnitine (C5)	0.06	0.04	0.02–0.11
Isovalerycarnitine (C5)	**0.29**	0.14	0.02–0.19
Valerylcarnitine (C5)	0.01	0.00	0–0.03
Tiglylcarnitine (C5:1)	0.00	0.00	0–0.03
3‐Methylcrotonylcarnitine (C5:1)	0.00	0.00	0–0
3‐Hydroxyisovalerylcarnitine (C5OH)	0.01	0.00	0–0.09
Glutarylcarnitine (C5DC)	**0.24**	**0.32**	0.01–0.06
Hexanoylcarnitine (C6)	**0.59**	**0.52**	0.01–0.09
Octanoylcarnitine (C8)	**1.4**	**3.43**	0.03–0.28
Decanoylcarnitine (C10)	**2.4**	**3.93**	0.03–0.4
Decenoylcarnitine (C10:1)	**0.27**	**0.62**	0.01–0.12
Dodecanoylcarnitine (C12)	**0.77**	**0.79**	0.01–0.15
Dodecenoylcarnitine (C12:1)	**0.21**	0.08	0–0.12
Tetradecanoylcarnitine (C14)	**0.62**	**0.15**	0.01–0.05
Tetradecenoylcarnitine (C14:1)	**0.91**	**0.27**	0.01–0.18
Tetradecadienoylcarnitine (C14:2)	**0.27**	**0.10**	0–0.8
3‐Hydroxytetradecanoylcarnitine (C14OH)	0.00	0.01	0–0.04
3‐Hydroxytetradecenoylcarnitine (C14:1OH)	0.00	0.01	0–0.06
Hexadecanoylcarnitine (C16)	**0.86**	**0.20**	0.03–0.13
3‐Hydroxyhexadecanoylcarnitine (C16OH)	0.00	**0.02**	0–0.01
3‐Hydroxyhexadecenoylcarnitine (C16:1OH)	0.00	0.01	0–0.01
Hexadecadikarboxylcarnitine (C16DC)	0.00	Not measured	0–0.05
Octadecanoylcarnitine (C18)	**0.55**	0.05	0.01–0.07
Octadecenonylcarnitine (C18:1)	**1.4**	**0.31**	0.04–0.29
Octadecadienoylcarnitine (C18:2)	**1.1**	0.12	0.02–0.23
3‐Hydroxyoctadecanoylcarnitine (C18OH)	0.00	**0.01**	0–0
3‐Hydroxyoctadecenoylcarnitine (C18:1OH)	0.01	0.00	0–0.01
Estimated total carnitine	25	49	22–60
C0/(C16 + C18)	**7**	127	126–842
(C16 + C18:1)/C2	**1.2**	**0.09**	0.01–0.06
Free/total	**41%**	**64%**	65%–87%

*Note:* Bold indicates abnormal values.

Abbreviations: mo.: months; Ref. range: reference range, from Metabolic Lab, Department of Genetics, Copenhagen University Hospital Rigshospitalet, Denmark; Treat.: treatment.

After 2 years of treatment, repeat muscle biopsy showed a myofiber pattern suggestive of less and more dispersed lipid that has a less disruptive effect on the ultrastructure of the myofiber (Figure 2). The patient has experienced great improvement with the treatment, however, still suffers from fatigue and has not regained his pre‐disease physical strength.

## 3 Discussion

We present a late‐onset MADD case with rapid physical deterioration resulting in almost complete loss of ambulation who presented uncommonly late in life. Diagnosing MADD displays numerous challenges as it is a rare disease and patients present with heterogenous symptoms and a great variation in age at symptom‐onset. Also, symptoms in MADD can resemble other disorders, such as riboflavin deficiency [8]. In this case, a metabolic disorder was not suspected at first. Therefore, not all standard diagnostic tests were carried out, such as urine analysis to assess organic acid excretion and blood tests to determine plasma amino acid levels along with all relevant metabolites. In this patient, blood glucose and plasma amino acid levels were normal. Ketones and ammonia levels were not assessed, nor were riboflavin levels measured. The above‐mentioned tests are important tools for establishing a correct and timely diagnosis, and we acknowledge that particularly the measurement of organic acids in urine samples could have facilitated the diagnostic process. However, in this case, the acylcarnitine‐profile, muscle biopsy and FDG PET/CT findings pointed us in the right direction, ultimately confirming the diagnosis through RNA sequencing. The synonymous variant p.(Leu299=) was first predicted as likely benign, but was ultimately associated with the ETFDH deficiency in combination with the p.(His588Arg) variant. Transcript level analysis was necessary to properly predict the effect of this synonymous variant.

In some cases of MADD, a triggering factor is found [9]. The disease trigger in this case was most likely a substantial increase of fat intake in the patient's diet that has been seen in a few other cases [10, 11], which similarly caused rapid deterioration. The patient never liked eating fatty food and had avoided it most of his life up until a new life partner started serving it. During a period with influenza‐like symptoms, the patient attempted to minimize his symptoms with a self‐discovered anti‐inflammatory diet partly consisting of a large amount of fish oil, which only worsened his symptoms and initiated his disease deterioration. The low level of functional ETFDH protein remaining in the patient might not have been sufficient to handle a sudden diet change, thereby triggering the disease onset.

This case highlights the need for increased awareness of rare disorders, such as MADD, among clinicians. Even though the standard diagnostic workup includes acylcarnitine profile, urine analyses, genetic testing and muscle biopsy, there is a chance that a patient with a similar rapid deterioration and unintended weight loss might have a 2‐[^18^F] FDG PET/CT performed to rule out malignancy/paraneoplasia. 2‐[^18^F] FDG PET/CT scans are well suited to diagnose occult malignancy as tumor cells often have an upregulated glucose metabolism [12]. In this case, the scan revealed a pathologically high glucose uptake in the patient's weak muscles. This finding is suggestive of a lipid storage myopathy, like MADD, in which patients cannot utilize fat properly within their skeletal muscles. High muscle glucose uptake can therefore also be suspected in other lipid storage myopathies or in autoimmune myositis such as polymyositis where glucose uptake is high due to inflammation [13]. Although not the sole tool of diagnostics in this patient, the 2‐[^18^F] FDG PET/CT scan could further support the existence of a lipid storage myopathy. RNA sequencing is, however, ultimately needed to confirm the diagnosis.

The use of 2‐[^18^F] FDG PET/CT for patients with metabolic myopathy is not well‐described in the literature with only three cases reported [14, 15, 16]. Similar to this study, the scan was used to rule out a paraneoplastic syndrome in these cases, but ultimately revealed a metabolic myopathy with only one case confirming a lipid storage myopathy. It is also important to emphasize that the late‐onset might never have materialized if the patient had not started eating fatty food, as the basal level of ETFDH apparently was sufficient to sustain a normal active life with low‐fat diet until the patient's diet changed.

The patient had an excellent treatment response with improvements in line with other reports [3, 4, 9], however, still suffers from fatigue. The high responsiveness to riboflavin treatment highlights the importance of diagnosing patients with MADD in a timely manner. Despite the great clinical treatment response, the posttreatment muscle biopsy and acylcarnitine profile were only improved but still abnormal. One case story shows that after several years, the muscle biopsy can reveal close to normal tissue [17], however in our patient, his muscle tissue was not completely restored after 2 years.

We learned from this case that MADD can present with a rapid onset late in life mimicking a paraneoplastic condition, and that in such a scenario a 2‐[^18^F] FDG PET/CT scan, showing diffuse and symmetric 2‐[^18^F] FDG uptake in skeletal muscle, can be valuable to direct the suspicion toward a lipid storage myopathy, such as MADD.

## Author Contributions

**Astrid Høj:** investigation, visualization, writing – original draft. **Sonja Holm‐Yildiz:** conceptualization, investigation, visualization writing – review and editing. **Thomas Krag:** conceptualization, investigation, supervision, visualization, resources, project administration, writing – review and editing. **Danijela Dejanovic:** investigation, resources, writing – review and editing. **Thomas van Overeem Hansen:** investigation, resources, writing – review and editing. **Morten Dunø:** investigation, resources, writing – review and editing. **Mette Cathrine Ørngreen:** investigation, writing – review and editing. **John Vissing:** conceptualization, supervision, validation, resources, writing – review and editing. **Nicoline Løkken:** conceptualization, investigation, supervision, visualization, writing – review and editing.

## Ethics Statement

The authors have nothing to report.

## Consent

The patient has signed a patient consent statement.

## Conflicts of Interest

The authors declare no conflicts of interest.

## Data Availability

The data that support the findings of this study are available on reasonable request from the corresponding author, Astrid Høj. The data are not publicly available due to the privacy or ethical restrictions of the research participants.
